# Fecal Microbiota Transplants Annually and Their Positive Clinical Impact

**DOI:** 10.14309/ctg.0000000000000247

**Published:** 2020-10-27

**Authors:** Lamia Mamoon, Scott W. Olesen

**Affiliations:** 1 OpenBiome, Cambridge, Massachusetts, USA.

## Abstract

**INTRODUCTION:**

Although fecal microbiota transplantation (FMT) is a recommended, clinically efficacious, and cost-effective treatment for recurrent *Clostridioides difficile* infection (CDI), the scale of FMT use in the United States is unknown.

**METHODS:**

We developed a population-level CDI model.

**RESULTS:**

We estimated that 48,000 FMTs could be performed annually, preventing 32,000 CDI recurrences.

**DISCUSSION:**

Improving access to FMT could lead to tens of thousands fewer *C. difficile* episodes per year.

## INTRODUCTION

*Clostridioides difficile* infection (CDI), the most common hospital-acquired infection in the United States, is a major public health threat, with more than 450,000 US cases in 2017 (1). Aside from severe diarrhea and other associated health conditions, recurrent CDI can lead to social isolation, humiliation, difficulty keeping a job because of days lost from work and an inability to leave the home, and emotional trauma (2). In 2017, *C. difficile* was estimated as contributing $1 billion to US healthcare costs (3). Approximately 10%–30% of primary patients with CDI suffer a recurrence of CDI after standard first-line antibiotic therapy, typically vancomycin or fidaxomicin (4,5). Of patients suffering a first recurrence, approximately 40% recur again (5), and some patients endure months or years in a cycle of disease, antibiotic therapy, remission, and recurrence (6).

Fecal microbiota transplantation (FMT), the transfer of stool from a healthy donor into the gastrointestinal tract of the patient, is a medically recommended treatment option for multiply recurrent CDI (4,7) permitted by the US Food and Drug Administration (8). Evidence from clinical trials suggest that FMT prevents further recurrences in approximately 80% of patients compared with 20%–50% for standard antibiotic therapy (8). Cost effectiveness studies have generally concluded that FMT is superior to standard antibiotic therapy for multiply recurrent CDI (9), and stool banks can relieve individual providers from the logistical complexities of screening donors and manufacturing FMT material (8,10).

Despite the emergence of FMT as a medically recommended and likely cost-effective treatment option for multiply recurrent CDI, and despite the characterization of FMT's clinical efficacy in multiple trials, there are, to our knowledge, no estimates of the total number of FMTs performed annually in the United States, nor of the number of CDI episodes prevented by the use of FMT. To address this gap, we developed a high-level, population-level mathematical model and adapted a recent cost effectiveness model (5) that accounts for patients' course through multiple CDI recurrences and the role that use of FMT could play in preventing further CDI recurrences. Using this model, we make preliminary estimates of the potential and realized the scale and clinical benefit of FMT.

## METHODS

The details of the model are in the Supplemental Information, (see Supplementary Digital Content 1, http://links.lww.com/CTG/A406). Briefly, the model bins the entire US population as at-risk of CDI, or suffering a primary CDI episode, a first recurrence, a second recurrence, a third recurrence, and so on. After the approach of Rajasingham et al. (5), in the model, some proportion of multiple recurrent patients with CDI (i.e., those suffering second or later CDI recurrence or equivalently a third or later overall CDI episode) are treated with FMT. We call this proportion the “coverage” of FMT. By scaling the coverage from 0% up through 100%, the model estimates the maximum number of FMTs potentially performed annually in the United States and the effects of altered coverage on the number of CDI episodes.

Of the model's 6 input parameters (see Table 1, Supplementary Digital Content 1, http://links.lww.com/CTG/A406), 4 were adapted from Rajasingham et al. (5) and 2 were drawn from other literature (1,6). The model had 3 outcomes: the annual number of FMTs performed, the proportion of CDI episodes that are multiple recurrent episodes, and the number needed to treat (see Table 2, Supplementary Digital Content 1, http://links.lww.com/CTG/A406). In this context, the number needed to treat is the average number of FMTs required to prevent 1 CDI episode.

To account for uncertainty in the input parameters, we drew 1 million bootstrap replicates from distributions around the parameter point estimates (see Table 1, Supplemental Information, Supplementary Digital Content 1, http://links.lww.com/CTG/A406). To evaluate the sensitivity of each outcome to the value of each input parameter, we varied each input parameter by 10% around the point estimate and evaluated the fractional change in each outcome.

All simulations and analyses were performed in R (version 3.6.0) (11). The code is available at DOI 10.5281/zenodo.3743343 (https://zenodo.org/record/3743343).

## RESULTS

**Figure 1.**
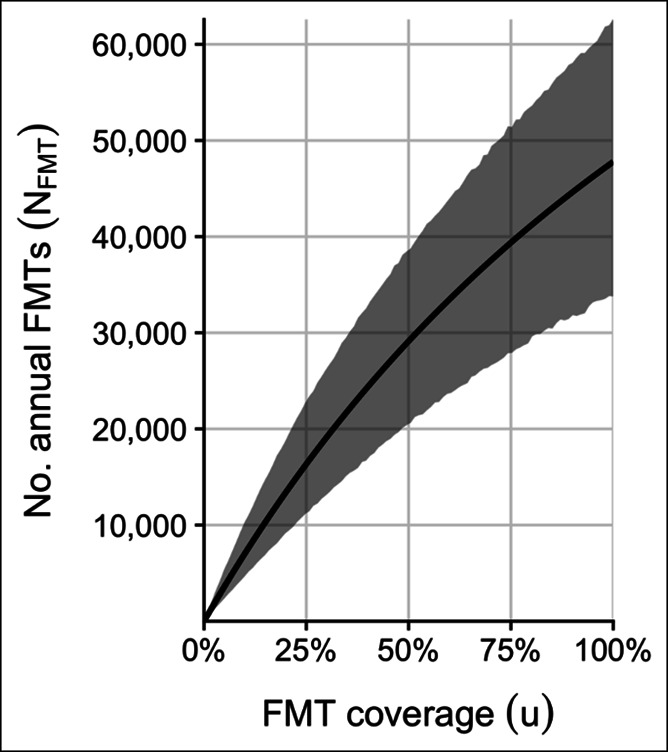
FMT coverage and the annual number of FMTs in the United States. Solid line shows the model's point estimate of the number of FMTs performed annually in the United States as a function of FMT coverage (i.e., the proportion of multiply recurrent CDI cases treated with FMT). Gray region shows 95% quantiles over the 1 million bootstrap variates. CDI, *Clostridioides difficile* infection; FMT, fecal microbiota transplantation.

The model estimated that if FMTs were being used to treat all multiple recurrent CDI cases, then 48,000 FMTs (95% confidence interval [CI] 34,000–62,000) would be performed annually in the United States (see Table 2, Supplementary Digital Content 1, http://links.lww.com/CTG/A406). The predicted number of FMTs depends strongly on the proportion of multiple recurrent cases treated with FMT (Figure 1, see Table 3, Figure 1, Supplementary Digital Content 1, http://links.lww.com/CTG/A406). For example, at 14% coverage, only 10,000 FMTs (95% CI 6,700–14,000) are performed. The proportion of CDI episodes that are multiple recurrent (i.e., third or later episodes) was mostly robust to coverage (see Tables 2 and 3, Supplemental Figure 1, Supplementary Digital Content 1, http://links.lww.com/CTG/A406). At 0% coverage, the model estimated this proportion at 16% (95% CI 11%–24%); at 100% coverage, 10% (95% CI 7%–13%). The estimated number of FMTs required to prevent 1 CDI recurrence was 1.5 (95% CI 0.6–4.7) and was very robust to the coverage level (see Tables 2 and 3, Figure 1, Supplementary Digital Content 1, http://links.lww.com/CTG/A406).

In a sensitivity analysis (see Table 3, Figure 2, Supplementary Digital Content 1, http://links.lww.com/CTG/A406), the number needed to treat was strongly dependent on the probability of recurrence when using standard therapy: for a 10% increase in the recurrence rate on standard therapy, this outcome declines by 28%. The second strongest dependence in the model was the total number of FMTs on the total number of CDI cases: for a 10% increase in total CDI cases, the number of FMTs increases by 10%.

## DISCUSSION

A population-wide model of the role of FMT in treating CDI recurrences estimated that 10%–15% of CDI cases are multiple recurrent, translating to as many as 48,000 FMTs in the United States annually. The model also estimated that for every 1.5 FMTs performed, 1 CDI episode is prevented. Thus, the full use of FMT could prevent as many as 32,000 CDI recurrences every year.

A strength of this model was its evaluation of the population-wide effect of FMT on CDI recurrences. Although clinical trials address the merit of FMT for the treatment of individual patients, and cost effectiveness studies address the facility-level economics of FMT, this model estimated the scale and clinical impact of this novel therapy. We expect that this model could serve as a scaffold for future refinements and improved estimates.

However, this analysis is subject to important limitations. First, as with any mathematical model, the accuracy of the results depends on the validity of the model design and the accuracy of the parameter estimates. Furthermore, the model makes simplifying assumptions about important clinical factors such asthe exact antibiotic regimens used and the route of FMT delivery. It therefore does not account for the fact that the use of more effective antibiotics would decrease FMT's impact, whereas the use of more effective routes of FMT delivery would increase its impact (see Table 3, Supplementary Digital Content 1, http://links.lww.com/CTG/A406).

Second, we considered only a single type of CDI, multiple recurrent disease, and a single health outcome, the number of CDI episodes. Emerging evidence suggests that FMT reduces the risk of septicemia in recurrent patients with CDI (12) and may reduce mortality when used as treatment for severe and fulminant CDI (8). FMT is also an experimental therapy for dozens of disease indications (8,13). Thus, future studies may need to account for multiple positive outcomes of FMT. Conversely, FMT has important safety risks. Known risks include the transfer of pathogens and complications associated with the delivery procedure, exemplified by the transmission of drug resistant *Escherichia coli* via FMT (14). Theoretical risks include the transfer of potentially microbiome-mediated diseases (8,10).

Third, the model estimated FMT's potential for preventing CDI recurrences only for the patients treated with FMT. However, preventing CDI episodes likely has a population-level benefit: fewer CDI episodes in one patient presumably means fewer transmissions and so fewer primary episodes in other individuals (15). Extending the model to include these population-level effects is possible but will require estimating the total size of the at-risk population and stratifying it by different transmission risks.

Fourth, the model does not distinguish between patient populations. The estimates for the number needed to treat and the number of CDI episodes prevented are therefore only valid as high-level, population-wide estimates. They may not be applicable for guiding the care of a particular patient or patient population in a particular setting. Stratifying patient populations by disease type, comorbidities, and setting will be important for balancing estimates of FMT's benefits against its risks.

Finally, we estimated the total possible number of avoided recurrences, but the public health impact of FMT depends on the actual number of FMTs performed. OpenBiome, a nonprofit stool bank and likely the largest single FMT material producer in the United States, shipped approximately 11,000 FMT preparations in 2019, setting an approximate lower bound for the number of FMTs performed in the United States. If only 11,000 FMTs are performed, then FMT coverage could be as low as 15%, and as many as 25,000 preventable CDI recurrences are not prevented (48,000–11,000 episodes/1.5 number needed to treat). We expect that private stool banks and patient-directed procedures account for a substantial number of annual FMTs, but we are not aware of any published estimates of those contributions to the total number of annual FMTs. To add further complexity, there could be simultaneous undertreatment and overtreatment: patients who qualify for FMT may not receive it, and patients for whom FMT is not indicated may be receiving it. Without more transparency around FMT practice, these questions remain topics for speculation only.

Overall, FMT has the potential for enormous positive clinical impact, preventing tens of thousands of CDI episodes every year. The FDA's policy of enforcement discretion, permitting the use of FMT to treat multiple recurrent CDI without an Investigational New Drug Application, has enabled the widespread use of this therapy (16). Stool banks have also played a key role in expanding safe access to FMT material that can be consistently screened in accordance with international guidelines (10). We encourage other stool banks to publish the number of FMT treatments they manufacture and hospitals to publish the number of FMTs they perform so that medical societies and regulators can better understand what barriers remain to widespread safe access to FMT and more informedly balance FMT's risks against its benefits.

## CONFLICTS OF INTEREST

**Guarantor of the article:** Scott W. Olesen, PhD.

**Specific author contributions:** L.M. conceived of the work and wrote the first draft. S.W.O. performed the analysis and revised the manuscript.

**Financial support:** OpenBiome.

**Potential competing interests:** None to report.

**Study Highlights**

**WHAT IS KNOWN**

✓ Fecal microbiota transplantation is an important treatment option for *Clostridioides difficile* infection.
✓ Stool banks play a key role in supplying FMT material.

**WHAT IS NEW HERE**

✓ A first estimate of the total number of FMTs performed in the United States.
✓ A first estimate of the total clinical impact of FMT.

**TRANSLATIONAL IMPACT**

✓ Improving access to FMT could lead to tens of thousands fewer *C. difficile* episodes per year.

## Supplementary Material

SUPPLEMENTARY MATERIAL
